# FRET biosensor allows spatio-temporal observation of shear stress-induced polar RhoGDIα activation

**DOI:** 10.1038/s42003-018-0232-2

**Published:** 2018-12-10

**Authors:** Shuai Shao, Xiaoling Liao, Fei Xie, Sha Deng, Xue Liu, Tapani Ristaniemi, Bo Liu

**Affiliations:** 1 School of Biomedical Engineering, Dalian University of Technology, Liaoning IC Technology Key Lab, 116024 Dalian, China; 2 Faculty of Information Technology, University of Jyväskylä, 40014 Jyväskylä, Finland; 3 Biomaterials and Live Cell Imaging Institute, Chongqing University of Science and Technology, 401331 Chongqing, China

**Keywords:** Fluorescence imaging, Cell polarity, RHO signalling

## Abstract

Rho GDP-dissociation inhibitor α (RhoGDIα) is a known negative regulator of the Rho family that shuts off GDP/GTP cycling and cytoplasm/membrane translocation to regulate cell migration. However, to our knowledge, no reports are available that focus on how the RhoGDIα-Rho GTPases complex is activated by laminar flow through exploring the activation of RhoGDIα itself. Here, we constructed a new biosensor using fluorescence resonance energy transfer (FRET) technology to measure the spatio-temporal activation of RhoGDIα in its binding with Rho GTPases in living HeLa cells. Using this biosensor, we find that the dissociation of the RhoGDIα-Rho GTPases complex is increased by shear stress, and its dissociation rate varies with subcellular location. Moreover, this process is mediated by membrane fluidity, cytoskeleton and Src activity, which indicates that the regulation of RhoGDIα activation under shear stress application represents a relatively separate pathway from the shear stress-induced Rho pathway.

## Abstract (web summary)

Shuai Shao, Xiaoling Liao et al. present a new FRET biosensor for measuring the spatio-temporal activation of RhoGDIα upon binding Rho GTPases. They find that dissociation of the RhoGDIα-Rho GTPase complex is increased by shear stress and varies with subcellular location.

## Introduction

Cell migration is a complicated process regulated by physical and chemical factors, playing a significant role in diverse physiological and pathological events, especially in tumor metastasis^1^. Before cell migration can occur, the concentrations of relevant factors are distributed in a spatially asymmetric manner, referred to as cell polarity^2^. This distribution pattern indicates the direction for migration and tumor metastasis^3^. A crucial factor contributing to the establishment of cell polarity is the Rho-family GTPases, which regulate the formation of lamellipodia and rearrangement of the cytoskeleton^4^. Rho GDP-dissociation inhibitor α (RhoGDIα), also known as RhoGDI1, is the main member of the RhoGDI family, is expressed ubiquitously^5^ and participates in the Rho cycle between the GTP-bound (active state, on membrane) form and GDP-bound (inactive state, in cytoplasm) form^6^. The steady state of GDP-binding Rho GTPases in cytosol is associated with RhoGDIα forming a RhoGDIα-Rho GTPases complex. The complex translocates to the plasma membrane when activated by Rho guanine nucleotide exchange factors (Rho GEFs) and then the complex dissociates. After completing their functions, inactive Rho GTPases will be extracted from the membrane by RhoGDIα^7^.

To date, most work has considered RhoGDIα as a negative regulator to Rho GTPases merely, ignoring its own mechanism of activation^8,9^. In fact, inhibiting RhoGDIα expression could promote invasion and metastasis of breast cancer cells and trophoblast stem cells^10,11^, but overexpression in hepatoma cells has a similar effect^12,13^. Moreover, some reports have proved that RhoGDIα can be mediated by other molecules. For example, the ezrin-radixin-moesin protein family (ERM) can bind RhoGDIα directly to release Rho GTPases^14^, and plexin-B3, a cell surface receptor of Semaphorin 5A, can interact with RhoGDIα transiently to promote the extraction of Rac-GTP from RhoGDIα to the cytoplasm^15^. Some kinases can even phosphorylate several amino acid sites of RhoGDIα directly to affect the formation process of RhoGDIα-Rho GTPases complex^16,17^. These findings indicate that there should exist a regulating pathway to RhoGDIα directly, ignored but important and independent of Rho GTPases.

However, because Rho GTPases can exert their regulation on RhoGDIα^9^, and RhoGDIα can play its role only when it is combined with the Rho GTPases, which can be considered as RhoGDIα activation for its function of inhibiting Rho GTPases activation, the absence of an efficient tool makes it challenging to observe RhoGDIα activation in its binding with Rho GTPases in living cells. In this study, we designed a biosensor using fluorescence resonance energy transfer (FRET) and tested its ability to detect RhoGDIα and Rho GTPase binding levels in living cells while avoiding the effect of Rho GTPases. We constructed a spatiotemporal model of the binding degree distribution of the RhoGDIα-Rho GTPases complex in living HeLa cells, and analyzed the effects of different magnitudes of shear stress. We also describe the pathways of RhoGDIα binding with Rho GTPases in cell migration. The results show that RhoGDIα activation has a regulating method relatively independent of Rho GTPases, which is activated under shear stress and is influenced by cell membrane fluidity, microfilaments, and Src.

## Results

### The sl-RhoGDIα FRET biosensor reflects RhoGDIα activity

**Fig. 1:**
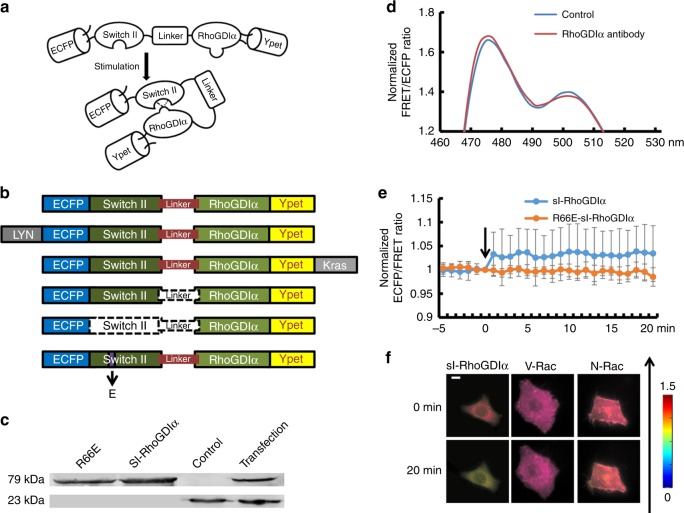
The verification experiments of sl-RhoGDIα biosensor. **a** The diagram of sl-RhoGDIα biosensor. **b** The structure of sl-RhoGDIα and derived biosensors. Dotted line means these parts do not exist in biosensor structure. **c** Western blot results at 23 and 79 kDa. From left to right is shown the purified R66E-sl-RhoGDIα protein, sl-RhoGDIα protein, and the disruption product of cells from the control group without transfection and transfected with sl-RhoGDIα biosensor. **d** The emission spectrum of sl-RhoGDIα biosensor before and after RhoGDIα antibody stimulation. **e** The FRET efficiency time series of sl-RhoGDIα and R66E-sl-RhoGDIα biosensor with stimulation of RhoGDIα antibody. **f** The living cell images of sl-RhoGDIα (*n* = 11), V-Rac and sl-RhoGDIα (*n* = 8), N-Rac and sl-RhoGDIα (*n* = 7) upon shear stress. The direction of shear stress is from bottom to up as shown by the arrow. The scale bar is 10 μm

To monitor real-time change in RhoGDIα-Rho GTPases binding caused by the regulation of RhoGDIα, rather than by the Rho GTPases, a FRET-based biosensor, sl-RhoGDIα, was designed according to the fact that RhoGDIα molecular can bind with a switch II domain (Fig. 1a). A variant biosensor R66E-sl-RhoGDIα was constructed, in which the 66 Arg of switch II was mutated to Glu to prevent its binding with RhoGDIα, as a negative control to inhibit the combination of RhoGDIα and switch II (Fig. 1b). S-RhoGDIα (without linker) and nsl-RhoGDIα (without Switch II and linker) were also constructed as controls to demonstrate the necessity of switch II and the linker sequence in the biosensor (Fig. 1b). Proteins of biosensors were purified from BL21-competent cells in vitro, and RhoGDIα antibody was utilized (1:1000, ARHGDIA polyclonal antibody, ABclonal) to detect RhoGDIα (23 kDa) and sl-RhoGDIα biosensor (79 kDa) by western blot. As shown in Fig. 1c (Supplementary Figure 1), clear bands could be observed near the weight of 23 kDa in the disruption product of cells transfected with the biosensor or control group without transfection, while at a weight ~79 kDa clear bands were also detected in purified sl-RhoGDIα biosensor protein, R66E-sl-RhoGDIα biosensor protein and the disruption product of cells transfected with sl-RhoGDIα biosensor, but not in control group. The results showed its stable expression in both eukaryotic cells and colibacillus.

To verify the function of the biosensor, the fluorescence emission spectra between 450–530 nm of purified sl-RhoGDIα protein was measured upon excitation at 420 nm (SpectraMax M2, Molecular Devices). A lower energy transmission efficiency was observed after adding a specific RhoGDIα antibody (dilution 1:25) to inhibit the binding between RhoGDIα and switch II (Fig. 1d). Moreover, the FRET efficiency (emission ratio of 475 nm/515 nm) of purified proteins of sl-RhoGDIα and its negative control biosensor R66E-sl-RhoGDIα was also measured upon excitation at 420 nm for 20 min. An increase was observed upon RhoGDIα antibody application in sl-RhoGDIα, but not in R66E-sl-RhoGDIα (Fig. 1e). These results indicated the validity of the biosensor design for the binding between RhoGDIα and switch II, and also showed the specificity of sl-RhoGDIα biosensor for the detection of RhoGDIα activation.

To further verify the validity, specificity, and reversibility of the biosensor in vivo, 20 dyn cm^−2^ of shear stress was applied on biosensor-transfected cells as the stimulation. An obvious decrease of the FRET efficiency, indicating the separation of switch II from RhoGDIα under stimulation, was found in sl-RhoGDIα but not in R66E-sl-RhoGDIα (Fig. 1f, Supplementary Figure 2a and 2b). It was interesting that the decrease stopped when the shear stress was removed after application lasting for 5 min, and then the FRET efficiency increased slowly with time after the shear stress was removed (Supplementary Figure 2b and 2c). Obvious differences between control group and reverse test began to occur when shear stress had been removed for 26 min and remained (*p*_26min_ = 0.034, *p*_27min_ = 0.037, *p*_28min_ = 0.042, *p*_29min_ = 0.044, and *p*_30min_ = 0.041, Supplementary Figure 2c). However, the decrease of FRET efficiency disappeared when the cell with sl-RhoGDIα was co-transfected with V-Rac or N-Rac plasmid, both of which provided exogenous switch II domain although they can enhance or inhibit Rac activity, respectively (Fig. 1f). These findings reconfirmed that the sl-RhoGDIα biosensor could effectively and specifically test the RhoGDIα activation. In addition, 2 mmol per L NaOH was used to destroy the hydrogen bonds between RhoGDIα and switch II within the biosensor, which caused a remarkable decrease of the FRET efficiency in sl-RhoGDIα biosensor, but not so obvious in s-RhoGDIα, nsl-RhoGDIα or R66E-sl RhoGDIα (Supplementary Figure 2d). The results implied that the main cause of energy transfer was the combination of RhoGDIα with switch II by hydrogen bonds, and the linker between them was necessary to enhance the energy transfer efficiency. Cells with biosensors were also treated with GTP since GTP participates in RhoGDIα-Rho GTPases binding. Ten μmol per L of GTP causes the reduction of FRET efficiency in sl-RhoGDIα and a gentle decrease in R66E-sl-RhoGDIα, while no effect was found in s-RhoGDIα or nsl-RhoGDIα (Supplementary Figure 2e). It also proved that switch II was required for energy transfer and the linker improves its efficiency.

These results implied that the sl-RhoGDIα biosensor could effectively detect the change of FRET efficiency caused by RhoGDIα's combination with switch II through hydrogen bonds in both in vitro and living cells. Moreover, the transfer efficiency was specially affected by the alternation of RhoGDIα activity, but not Rho GTPases.

### Sublocation and magnitude of flow affect RhoGDIα activity

Since Rho GTPases existed in two different states, activated on the membrane and non-activated in the cytoplasm^18^, sl-RhoGDIα was designed to show the affinity of the RhoGDIα-Rho GTPases in the cytoplasm, while Kras- and Lyn-sl-RhoGDIα indicated the presence of the complex on the cell membrane, at the non-lipid raft regions or on lipid rafts. Images of cells transfected with sl-RhoGDIα, Kras-sl-RhoGDIα, and Lyn-sl-RhoGDIα biosensor, respectively, showed obvious local differences in living cells, proving that sl-RhoGDIα biosensor existed in the cytoplasm, and Kras/Lyn sequence linked the biosensor to the cell membrane as expected. Shear stress was applied for 30 min to ensure biosensors had long enough time to become steady. A shear stress of 5 dyn cm^−2^ led to a FRET ratio decrease of ~25% in sl-RhoGDIα (Supplementary Movie 1, Supplementary Figure 3a, Supplementary Table 1) and Kras-sl-RhoGDIα (Supplementary Movie 2, Supplementary Figure 3a, Supplementary Table 2). Thus, RhoGDIα activity in cytoplasm and non-lipid raft regions on membrane decreased similarly with shear stress application (Supplementary Table 3, *p*_Kras-Cyto_ = 0.618).

**Fig. 2:**
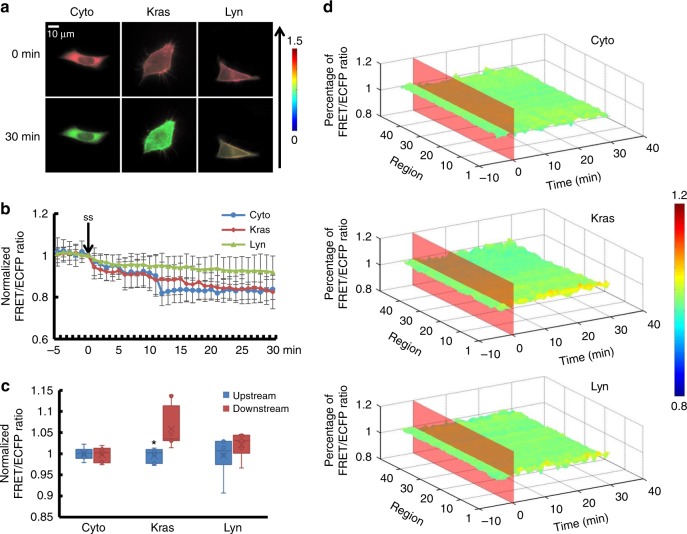
The affinity of RhoGDIα and Rho GTPases at different subcellular locations under 5 dyn cm^−2^ of shear stress. **a** Living cell images of three biosensors under 5 dyn cm^−2^ of shear stress. Cyto represents the biosensor sl-RhoGDIα (*n* = 6), which exists in the cytoplasm. Kras represents Kras-sl-RhoGDIα (*n* = 8) and Lyn represents Lyn-sl-RhoGDIα (*n* = 6). The arrow shows the direction of shear stress. The scale bar is 10 μm. **b** Effect of shear stress on the binding degree of RhoGDIα and Rho. **c** The FRET/ECFP ratio comparison of upstream to downstream, after normalization. The asterisk denotes that there is an obvious difference between upstream and downstream. **d** The binding degree distribution of RhoGDIα and Rho. The FRET ratio percentage of each region overall is normalized before shear stress application. The dissociation of the RhoGDIα-Rho complex is inhibited downstream along the flow direction

**Fig. 3:**
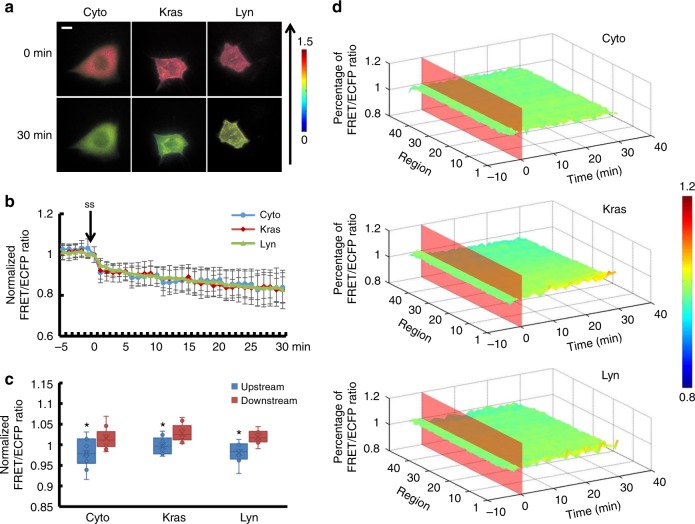
The affinity of RhoGDIα and Rho GTPases at different subcellular locations under 20 dyn cm^−2^ of shear stress. **a** Living cell images of three biosensors under 20 dyn cm^−2^ of shear stress. Biosensors are indicated as in Fig. 2, Cyto (*n* = 10), Kras (*n* = 9) and Lyn (*n* = 8). The arrow shows the direction of shear stress. The scale bar is 10 μm. **b** Binding degree of RhoGDIα and Rho as a function of shear stress. **c** The FRET/ECFP ratio comparison of upstream to downstream, after normalization. The asterisk denotes that there is an obvious difference between upstream and downstream. **d** The binding degree distribution of RhoGDIα and Rho. The FRET ratio percentage of each region overall is normalized before shear stress application. The dissociation of RhoGDIα-Rho GTPases complex is inhibited downstream along the flow direction

**Fig. 4:**
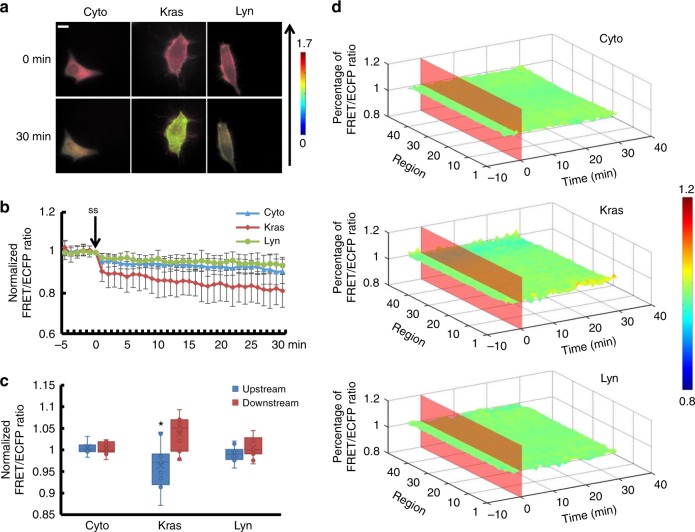
The affinity of RhoGDIα and Rho GTPases at different subcellular locations under 40 dyn cm^−2^ of shear stress. **a** Living cell images of three biosensors under 40 dyn cm^−2^ of shear stress. Biosensors labeled as in Fig. 2, Cyto (*n* = 11), Kras (*n* = 11) and Lyn (*n* = 11). The arrow shows the direction of shear stress. The scale bar is 10 μm. **b** Binding degree of RhoGDIα and Rho as a function of shear stress. **c** The FRET/ECFP ratio comparison of upstream to downstream, after normalization. The asterisk denotes that there is an obvious difference between upstream and downstream. **d** The binding degree distribution of RhoGDIα and Rho. The FRET ratio percentage of each region overall is normalized before shear stress application. The dissociation of RhoGDIα-Rho GTPases complex is inhibited at downstream along the flow direction

H owever, in Lyn-sl-RhoGDIα (Supplementary Movie 3), RhoGDIα activity remained relatively unchanged after flow application and differed from sl-RhoGDIα and Kras-sl-RhoGDIα (Fig. 2b, *p*_Lyn-Cyto_ = 0.012, *p*_Lyn-Kras_ = 0.007). Moreover, the differences between these biosensors disappeared when the magnitude of shear stress rose to 20 dyn cm^−2^, which indicated similar responses of RhoGDIα activity, decreasing about 20% at different positions (Fig. 3b, Supplementary Movie 4–6, Supplementary Figure 3b, Supplementary Table 3, *p*_Lyn-Cyto_ = 0.820, *p*_Lyn-Kras_ = 0.880, *p*_Kras-Cyto_ = 0.745). For a shear stress of 40dyn cm^−2^, the dissociation of RhoGDIα and Rho GTPases in the cytoplasm and lipid rafts was less than the other two flow patterns, but it remained remarkable in non-lipid raft regions (Fig. 4b, Supplementary Movie 7–9, Supplementary Figure 3d).

All three biosensors showed that the RhoGDIα-Rho GTPases complex dissociated under shear stress, and the distribution of complex dissociation varied with magnitude of the shear stress. With the Kras-sl-RhoGDIα biosensor, 5 dyn cm^−2^ of shear stress caused an obvious difference (*p* = 0.015) between upstream (the edge facing the flow) and downstream measures (the edge opposite to the upstream along the direction of flow), with higher activity of RhoGDIα downstream in non-lipid raft regions on the cell membrane. However, the corresponding activity in the cytoplasm of the overall region measured with the sl-RhoGDIα biosensor and in lipid rafts measured by the Lyn-sl-RhoGDIα biosensor did not change (Fig. 2c, d). In addition, RhoGDIα activity showed polarization (*p*_Cyto_ = 0.004, *p* _Kras_ =0.026, *p*_Lyn_ = 0.034) for all three biosensors under 20 dyn cm^−2^ of shear stress (Fig. 3c, d). Under 40 dyn cm^−2^ of shear stress, the difference disappeared for sl-RhoGDIα and Lyn-sl-RhoGDIα (*p* = 0.001), but remained for Kras-sl-RhoGDIα (Fig. 4c, d). These results indicated that RhoGDIα-Rho GTPases had complex dissociation patterns under shear stress that were affected by the magnitudes of flow, and that the polarization of RhoGDIα activity was more obvious on the cell membrane, especially on non-lipid raft regions.

### Membrane fluidity affects RhoGDIα activity with laminar flow

**Fig. 5:**
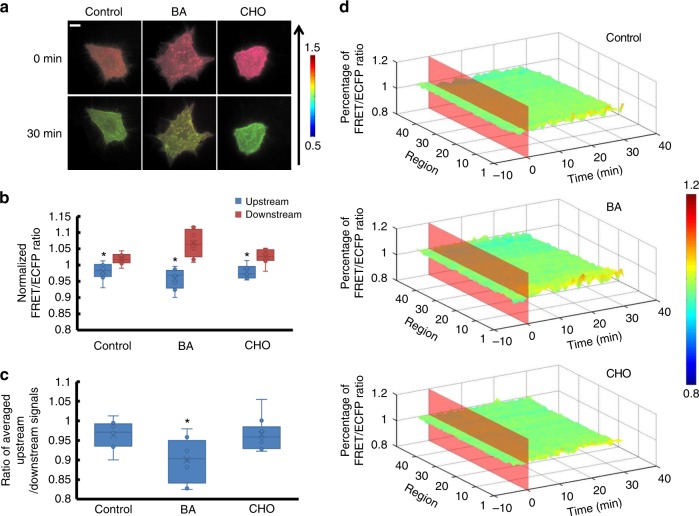
The affinity of RhoGDIα and Rho GTPases under shear stress is affected by membrane fluidity. **a** Living cell images of Lyn-sl-RhoGDIα biosensor under 20 dyn cm^−2^ of shear stress with 45 mmol per L benzol alcohol (BA, *n* = 8) or 0.1 mmol per L of cholesterol (CHO, *n* = 10). The scale bar is 10 μm. **b** The FRET/ECFP ratio comparison of upstream to downstream, after normalization. **c** The ratio of averaged upstream/downstream for the control group and the BA/CHO group. The asterisk denotes that there is an obvious difference between upstream and downstream. **d** The binding degree distribution of RhoGDIα and Rho GTPases when membrane fluidity is changed. The FRET ratio percentage of each region overall is normalized before shear stress application. The dissociation of the RhoGDIα-Rho GTPases complex is inhibited more downstream along the flow direction when membrane fluidity is enhanced by BA. The asterisk denotes that there is an obvious difference compared to control group

As shown above, shear stress-induced RhoGDIα activity change on the cell membrane had a more obvious polarity, so the Lyn-sl-RhoGDIα biosensor was applied in the following experiments for testing the effect of membrane fluidity. Since membrane fluidity was the most notable feature of cell membrane and inseparable from lipid rafts, benzol alcohol (BA) pre-incubation at 45 mmol per L for 15 min was used to enhance membrane fluidity^19^. The effect of membrane fluidity on RhoGDIα activity under 20 dyn cm^−2^ of shear stress was then assessed. Affinity polarity became stronger compared to the control group (*p*=0.015, Fig. 5b–d), while overall activity still decreased similarly to the control group (Supplementary Movie 10, Supplementary Figure 4a, Supplementary Table 4, *p*_BA-Control_ = 0.722). When cells were treated with cholesterol (CHO) at 0.1 mmol per L for 3 h to reduce membrane fluidity, RhoGDIα activity decreased about 30% upon laminar flow application, which was more significant than the control (Supplementary Figure 4a and Supplementary Movie 11). Although polarity still existed after CHO pretreatment, no obvious difference could be found on comparing this treatment to the control group (Fig. 5b–d, Supplementary Table 4, *p*_CHO-Control_ = 0.007). Thus, enhancing membrane fluidity aggravated the polarization of shear stress-induced RhoGDIα activity, while inhibiting membrane fluidity only affected the activity.

### Cytoskeleton participates in RhoGDIα polarity upon shear stress

**Fig. 6:**
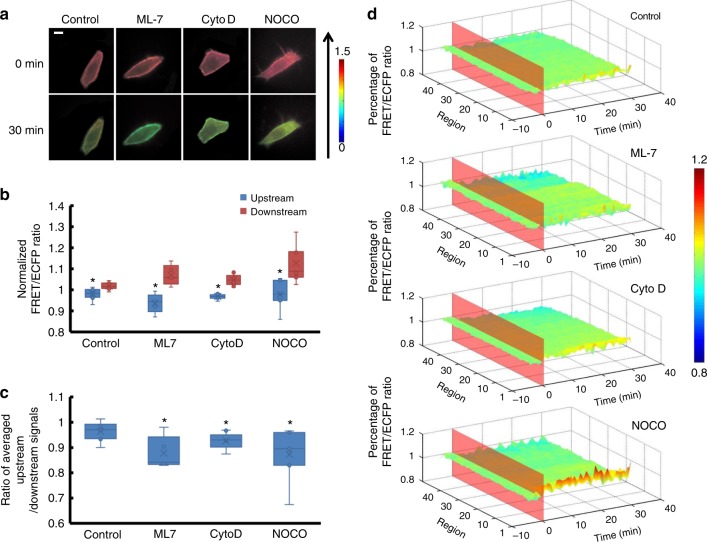
The affinity of RhoGDIα and Rho GTPases under shear stress is affected by cytoskeleton. **a** The living cell images of Lyn-sl-RhoGDIα biosensor under 20 dyn cm^−2^ of shear stress treated with 5 μmol/l of ML-7(*n* = 5), 2 μmol per L of Cytochalasin D (CytoD, *n* = 9), or 1 μmol per L of nocodazole (NOCO, *n* = 7). The scale bar is 10 μm. **b** The FRET/ECFP ratio comparison of upstream to downstream after normalization. **c** The averaged upstream/downstream ratio in the control group and in BA/CHO groups. The asterisk denotes that there is an obvious difference between upstream and downstream. **d** The binding degree distribution of RhoGDIα and Rho GTPases is enhanced downstream along the flow direction when cytoskeleton is disturbed. The FRET ratio percentage of each region overall is normalized before shear stress application. The asterisk denotes that there is an obvious difference compared to the control group

Since the cytoskeleton was closely related to the cell membrane, it seemed that the cytoskeleton would affect the RhoGDIα-Rho GTPases complex. To test this, cells were pre-treated with different drugs to depolymerize different components of the cytoskeleton before 20 dyn cm^−2^ of shear stress was applied. ML-7 was an inhibitor of myosin light chain kinase (MLCK) that could eliminate force transmissions only through microfilaments while the structure remains intact^19,20^. When microfilaments were treated with 5 μmol per L of ML-7 for 1 h before laminar shear stress application, more obvious RhoGDIα activity reduction, nearly 38%, under shear stress was observed (Supplementary Movie 12, Supplementary Figure 4b, Supplementary Table 4, *p*_ML7-Control_ = 0.005). The local activity distribution was more polarized (*p* = 0.007) compared to the control group (Fig. 6). Cytochalasin D (CytoD) pre-incubation at 2 μmol per L for 1 h to destroy the microfilament caused a similar effect on the RhoGDIα-Rho GTPases complex after 30 min of laminar shear stress application. The dissociation of the complex decreased to 35% and the polarity increased compared to the control group (*p* = 0.032; Fig. 6 and Supplementary Figure 4b and Supplementary Movie 13). This indicated that microfilaments participated in shear stress-induced RhoGDIα activation. However, compared to the control group, no significant change of RhoGDIα activity could be observed after application of 1 μmol per L of nocodazole (NOCO), a depolymerizing agent of microtubules, for 1 h before laminar shear stress was applied. However, RhoGDIα activity had a polarized distribution that was more noticeable than that of the control group when microtubules were destroyed (*p* = 0.025; Fig. 6d and Supplementary Movie 14). Thus, the cytoskeleton participated in the dissociation of the RhoGDIα-Rho GTPases complex under shear stress, and microfilaments played a more important role than microtubules.

### Shear stress-induced RhoGDIα polarity is related to Src

**Fig. 7:**
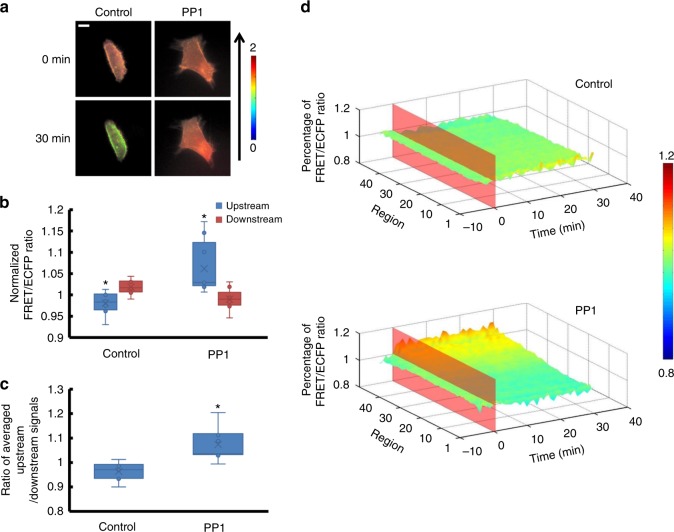
The affinity of RhoGDIα and Rho GTPases under shear stress is affected by Src. **a** Living cell images of Lyn-sl-RhoGDIα biosensor under 20 dyn cm^−2^ of shear stress with 50 mmol per L of the Src inhibitor PP1(*n* = 7). The scale bar is 10 μm. **b** The FRET/ECFP ratio comparison of upstream to downstream, after normalization. **c** Averaged upstream/downstream ratio for the control group and the PP1 group. The asterisk denotes that there is an obvious difference between upstream and downstream. **d** Binding degree distribution of RhoGDIα and Rho GTPases is enhanced at upstream regions along the flow direction when Src is inhibited. The FRET ratio percentage of each region overall is normalized before shear stress application. The asterisk denotes that there is an obvious difference compared to the control group

Since Src can phosphorylate RhoGDIα to inhibit RhoGDIα and Rho GTPases from forming complexes^21^, the effect of Src was tested by pre-treating cells with 50 mmol per L of Src inhibitor PP1 for 30 min^19^. The result clearly revealed that the RhoGDIα activity declined dramatically downstream when shear stress was applied (Fig. 7d, Supplementary Table 4, *p*_pp1-Control_ = 0.006), and the overall activity rose after laminar flow application, compared to the control group (Supplementary Movie 15, Supplementary Figure 4c).

### RhoGDIα activation is different on non-lipid raft regions

As the results revealed, the activity and polarity of RhoGDIα upon shear stress were related to membrane fluidity, cytoskeleton and Src, while the Kras-based biosensor performaned differently. Inhibiting Src activity can keep the complex of RhoGDIα and Rho GTPases dissociating at non-lipid raft regions upon shear stress (Supplementary Movie 21, Supplementary Figures 5a and 5d, *n*_pp1_ = 5), which accorded with the result of Lyn-based biosensor. However, with the same pretreatment to membrane fluidity and cytoskeleton, the overall activity of RhoGDIα showed no obvious difference compared to the control group (Supplementary Figures 5a, 5b and 5c, Supplementary Movie 17–20).

As to the affinity distribution of RhoGDIα and Rho GTPases at non-lipid raft regions, inhibiting Src activity caused a polarity exchange between upstream and downstream (Supplementary Figure 6), and increasing membrane fluidity promoted the polarity after shear stress application (*p* = 0.007, Supplementary Figure 7), which were consistent with the polar distribution on lipid rafts. However, the changes in microfilaments status caused by ML-7 and CytoD did not affect RhoGDIα polarity, while destroying microtubules by NOCO improved the polarity (Supplementary Figure 8).

## Discussion

Previous studies on RhoGDIα mainly focused on its regulation of Rho GTPases. Less attention has been given to how RhoGDIα is activated in the physiologic processes. The likely reason for this gap in the field was the lack of an adequate tool to detect uncoupled RhoGDIα in living cells without the interference of Rho GTPases. Wu et al. have reported a RhoGDIα-YFP biosensor that was co-transfected with CFP-ROP2 and CFP-ROP6 to form energy transfers in living cells, which could catch the interaction between RhoGDIα and ROPs^22^. Konstadinos provided another assay to visualize the control of Rac1 membrane targeting. Two different GFP-tagged Rac, co-expressed with RhoGDIα and without RhoGDIα, were transfected with MCherry into cells. When GFP-tagged Rac was transmitted to cell membrane, the FRET ratio between MCherry and GFP changed. By comparing the ratio difference between GFP-tagged RhoGDIα-Rac and GFP-tagged Rac, this assay can show the ability of RhoGDIα to inhibit the membrane-targeted translocation of Rac^23^. Hodgson attached a fluorescence protein pair to the N-terminus of Cdc42 with a binding antenna. The FRET ratio between two fluorescence proteins responded strongly only to the interaction with RhoGDIα, which can exhibit the spatio-temporal dynamics of the RhoGDIα-Cdc42 interaction^24^. However, these biosensors could only show the interaction of RhoGDIα with a single Rho family membrane, which is affected by the activation of Rho GTPases. Therefore, the change of RhoGDIα itself without the effect of Rho GTPases could not be tested, because of the conformational changes of Rho GTPases caused by its activation.

Based on FRET technology, a new biosensor is designed in this project to observe the binding of RhoGDIα and Rho GTPases, indicating the activity of RhoGDIα. The efficiency of energy transfer is related to the distance between fluorescence protein pairs, which is adjusted by RhoGDIα combining with switch II through hydrogen bonds. Destroying hydrogen bonds by alkaline or mutating switch II declines the efficiency, which confirms the conformational changes caused by combination within the biosensor and also proves that the principle of sl-RhoGDIα biosensor is effective.

The central structure of sl-RhoGDIα biosensor is switch II, a sequence shared by Rho GTPases that can combine with RhoGDIα through hydrogen bonds. The switch II domain within the biosensor prevents the potential effects of RhoGDIα overexpression on endogenous Rho GTPases. In living cells, the FRET efficiency of sl-RhoGDIα biosensor decreases after shear stress application and recovers slightly when the shear stress is removed. This trend of slow increasing after the shear stress removal implies that the FRET efficiency responding to stimulus is reversible. Besides, the energy transfer efficiency changed by spatial reconstruction should be accomplished only through RhoGDIα activation, but not switch II due to its stable characteristics^11^. This is proved by the co-transfection of sl-RhoGDIα with N-Rac or V-Rac. Although specific sites are mutated in N-Rac or V-Rac to inhibit or enhance Rac activity respectively^25,26^, a same inhibition of shear stress-induced FRET efficiency is observed in the biosensor, since both N-Rac or V-Rac can bind to the RhoGDIα within the sl-RhoGDIα biosensor in living cells. The RhoGDIα antibody experiment in vitro shows a similar testification in specificity. The antibody can bind to RhoGDIα specifically in the biosensor and block its switch II binding sites. In addition, the biosensor shows its reversibility in living cells and prefect stability in both vitro and living cells, thus providing a visual tool for exploring the mechanism of RhoGDIα regulation in its association with Rho GTPases. However, it should be noted that the sl-RhoGDIα biosensor indicates the alternation of spatio-temporal RhoGDIα activity responding to stimulus, not the real binding degree between RhoGDIα and Rho GTPases in living cells.

The current study shows that RhoGDIα separates from switch II when laminar flow is applied to the cell, which means that the RhoGDIα-Rho GTPases complex dissociates and the inhibition of RhoGDIα to Rho GTPases decreases under shear stress. This phenomenon accords with the fact that Rho GTPases are activated by shear stress^27^. In addition, this dissociation is not uniform along the direction of laminar flow. This finding is similar to the feature of shear stress-induced Rho GTPases activation, which typically shows a strong spatial pattern. It was verified that the family member RhoA activity peaks at the leading edge followed by Cdc42 and Rac^28^. Upon shear stress application, Rac1 is activated at the leading edge of cells along the flow direction^29–31^, and activated Cdc42 also polarizes at downstream regions^32^. Interestingly, RhoGDIα activation decreases more slowly at similar positions in the current work, indicating that negative regulation occurs in the process of RhoGDIα regulating Rho GTPases upon shear stress. Activated Rho GTPases assemble at downstream regions along the direction of shear stress, while the RhoGDIα-Rho GTPase complex binding at the same region is higher than in other regions. Paradoxically, a similar phenomenon has been observed in another work. RhoGDIα activated by the phosphorylation of Src exhibits lower affinity with Rho-GDP and translocates to the leading edge of cells where the GTP binding-Rho assemble^33^, which seems to show that RhoGDIα attempts to regulate Rho activation to a normal level. However, the mechanism detail is still unclear.

The RhoGDIα activity is strongly related to subcellular location and force magnitudes. The expression of activated Rac1 increases under low shear stress (5 dyn cm^−2^) compared to normal shear stress (20 dyn cm^−2^)^34^. As a negative regulator, the complex dissociation in cytoplasm experiences more under low-shear stress than normal or high-shear stress. The results conform to previous reports suggesting that low-shear stress would abate the dissociation of the RhoGDIα-Rho GTPases complex and release more Rho GTPases transformed to bind GTP^34^. However, RhoGDIα activity on the membrane appears to undergo eccentric changes. The activity of RhoGDIα in non-lipid raft regions is insensitive to force magnitudes. This is probably because after the dissociation of Rac1 or related Rho GTPases from RhoGDIα, they will be affected by some GTPase-activating proteins (GAPs) and then turn into non-activated status if they transfer to non-lipid raft regions^23^. It means that the key to keeping Rho GTPases non-activated at non-lipid raft regions should be GAPs, not RhoGDIα, and therefore the activity shown by the biosensor remains steady upon different levels of shear stress. However, the activity of RhoGDIα on lipid rafts decreases to a greater extent under normal than high- or low-shear stress. The difference may be attributable to the structure of lipid rafts and cytoskeleton. Lipid rafts are membrane domains that are enriched with cholesterol and certain saturated acyl lipids^35,36^. Those domains are dynamic and anchored by actin filaments. This anchoring of lipid rafts with actin filaments allows lipid rafts to move in a limited range^37^, and differences observed among force magnitudes might reflect the kinetic features of lipid rafts or actin filaments. Actin filaments are the basic intracellular traction force. Actin stress fibers gradually disappear in response to intermediate shear stress but increase with low- or high-shear stress^38,39^. There is a close relationship between actin stress fibers and Src, the important regulator to RhoGDIα activity, which helps actin stress fibers to mediate RhoGDIα. In our results, RhoGDIα activity on lipid rafts is promoted when actin filaments are increased by low- or high-shear stress (Supplementary Figure 4f), while destroying microfilaments with drugs aggravates dissociation of the RhoGDIα-Rho GTPases complex (Supplementary Figure 4b). It is possible that the dissociation of the RhoGDIα-Rho GTPases complex is regulated by shear stress depending on its magnitude, which causes actin filament reorganization and then affects Src polarity. The phenomenon is most prominent in lipid raft regions, probably because actin filaments link to lipid rafts directly.

The cell membrane isolates the cell from the external environment and transmits force in a polarized manner upon mechanical loading^40^. The cytoskeleton may sense and transmit mechanical force to specific sites of the cell, since many sites on the membrane can be coupled with actin microfilaments^41^. Shear stress can be sensed by the membrane initially and then transferred to the actin cytoskeleton directly by membrane deformation, and then transmitted through the actin cytoskeleton to activate subsequent signaling pathways^42,43^. Indeed, our results show that membrane fluidity and cytoskeleton affect shear stress-induced RhoGDIα-Rho GTPase complex formation and dissociation. This mediation is more obvious on lipid rafts, while to the complex at non-lipid raft regions, only the polarity of combination is impacted by the status of membrane fluidity or cytoskeleton. The overall activity of RhoGDIα at non-lipid raft regions has no distinct response when the membrane fluidity or cytoskeleton is interfered. Together with the results that the RhoGDIα activity at non-lipid raft region has no clear relationship with the shear stress magnitudes mentioned above, it seems that the RhoGDIα mediated by shear stress is on lipid rafts mainly, not at non-lipid raft regions. Interestingly, promoting membrane fluidity alone can change the affinity distribution of the RhoGDIα-Rho GTPases complex on both of non-lipid raft regions and lipid rafts, while inhibiting membrane fluidity has no effects. This may be because inhibiting membrane fluidity causes the positions of lipid rafts to become more fixed and thus it is more difficult for the polarity to be formed. However, it seems that RhoGDIα activity is not changed by membrane fluidity or cytoskeleton directly upon shear stress in our results.

Src localizes on the endosomal membranes as a type of non-receptor kinase. Shear stress can cause a polarized Src activation in endothelial cell at the edge facing the flow^19^. ß_3_ integrins are anchored to actin stress fibers and function as mechanosensors^44^. Its cytoplasmic tail recruits Src and Shp-1/2 to form a signaling complex, and then PKG II transforms to the ß_3_-Shp-Src complex to dephosphorylate Shp-1; Shp-1 activation courses Src phosphorylation to active Src in a non-uniform manner;^19,45^ Src-mediated RhoGDIα phosphorylation prevents the interaction and rebinding of membrane-associated Rho GTPases with RhoGDIα^21^. The polarizing activity of Src provides the spatial guide and regulates the dissociation of RhoGDIα and Rho GTPases through phosphorylating the former or maintains the complex in its original state. In the HeLa cells used for this study, when Src is specifically inhibited by PP1^19,46^, the distribution of RhoGDIα-Rho GTPases still generates a polarization transfer. This is probably because Src polarity is eliminated or weakened by PP1 at the upstream region, and then Src-mediated phosphorylation of RhoGDIα decreases accordingly at those regions. Furthermore, the ability of RhoGDIα to form a complex with Rho GTPases is enhanced dramatically at the location. Further evidence is that polarized Src activation is affected by actin filaments and membrane fluidity under laminar flow. Disruption of actin filaments may enhance Src polarized activation, causing more Rho GTPases dissociation from RhoGDIα. Benzyl alcohol-mediated enhancement of membrane fluidity could inhibit shear stress-induced Src polarity^19^. A similar conclusion is suggested from the result that the RhoGDIα-Rho GTPases complex separates more obviously when membrane fluidity is inhibited. Therefore, shear stress-induced dissociation of RhoGDIα and Rho could be attributed to Src activation changes that occur under shear stress.

As a regulatory factor of Rho GTPases, most researches have reported that the combination of RhoGDIα and Rho is based on Rho GTPases activation. However, some upstream molecules target RhoGDIα directly to alter its conformation, modify it, and change its location to affect downstream signaling pathways^47^. Those microfilaments play different roles in the binding of RhoGDIα and Rho GTPases mediated by shear stress on lipid rafts and non-lipid rafts, which was an unexpected result. Upon stimulation of growth factors, Src transports to lipid rafts from perinuclear regions and is activated in an actin-dependent manner, but activated without transmission at non-lipid raft regions with the help of microtubules^48^, indicating that Src activation is dependent on subcellular location. It may be the reason why microfilaments can affect RhoGDIα polarity on lipid rafts, but microtubules affect the polarity at non-lipid rafts. In a previous study, Rac1 was activated by shear stress directly by the force transmitting downstream with the help of the cell membrane and microtubules, but this has no relationship with Src^49^. In addition, Src activation is slower than Rac1^49^ upon shear stress application, which means that Src-mediated RhoGDIα activation would be much slower than Rac1. Based on these results, shear stress-induced Rac1 activation should have no direct connection with RhoGDIα activation. In addition, it was also found that the cell membrane sensed the pattern of flow and deformed to transmit force to active Rho GTPases on lipid rafts under shear stress through microtubules^27,50^, while active RhoGDIα was recruited through actin with the help of Src. Different cytoskeleton components mediate Rho GTPases and RhoGDIα activation when shear stress is applied. Therefore, it can be hypothesized that shear stress-induced RhoGDIα activation occurs in a different manner from Rho GTPases, although RhoGDIα is a negative regulator of Rho GTPases.

**Fig. 8:**
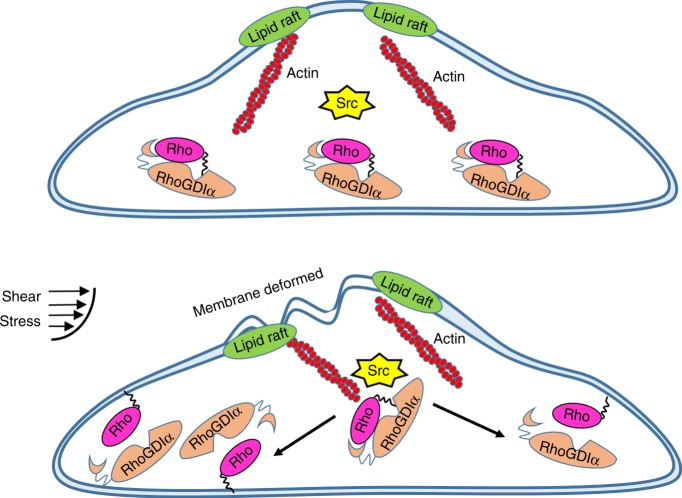
The proposed mechanism of shear stress induced-RhoGDIα activation

In this paper, a FRET biosensor that can measure the degree of binding for RhoGDIα and Rho in living cells is proposed, providing a useful visual tool to observe the activation of RhoGDIα in real-time without the interference of Rho GTPases. With results from the biosensor, a model of the regulation of the RhoGDIα-Rho GTPases complex and how RhoGDIα exerts its function under shear stress is built. The model can be simplified as follows (Fig. 8): the plasma membrane deforms when extracellular shear stress is applied to increase the cell membrane fluidity inhomogeneously, and then transfers the stress into intracellular forces, which are transmitted along actin filaments to the stress concentration point at the distal end through their contractions. Some mechanosensors such as ß_3_ integrins anchored to the actin stress fibers^44^ swing their tails and phosphorylate Src locally through ß_3_-Shp-Src complex;^19,45^ the polarizing activity of Src provides the spatial guide and regulates the dissociation of RhoGDIα and Rho GTPases through phosphorylating the former, or maintains the complex in its original state. This pathway is relatively independent, having no direct relationship with shear stress-induced Rho GTPase activation.

## Methods

### Design and establishment of sl-RhoGDIα-FRET biosensor

The biosensor, named sl-RhoGDIα, consists of a complete RhoGDIα sequence, a switch II sequence, a linker sequence, and ECFP/Ypet fluorescent protein pairs for FRET^51^ (Fig. 1b). Switch II is a common domain shared by Rho GTPases and can form contacts with RhoGDIα, which do not appear to create dramatic changes in its own conformation^11^. In the biosensor construction, the distance changes between switch II and RhoGDIα represent the affinity changes of RhoGDIα and Rho GTPases, which should be only caused by variation in RhoGDIα activity^11^. A linker sequence (GGSGGT) was designed between the RhoGDIα and switch II domain to provide a site for bending to improve the FRET efficiency. To demonstrate the necessity of switch II and the linker sequence, contrast biosensors with the switch II sequence only and without the switch II sequence or linker were also designed as s-RhoGDIα and nsl-RhoGDIα, respectively (Fig. 1b). Since the combination of switch II and RhoGDIα depends on hydrogen bonds between 185 Asp, 30 Ala, and 31 Pro in RhoGDIα and 66 Arg in switch II, the 66 Arg in switch II domain of sl-RhoGDIα was mutated to Glu in order to destroy hydrogen bonds between RhoGDIα and switch II^11^ to create a negative control biosensor, R66E-sl-RhoGDIα.

Utilizing the basic structure of sl-RhoGDIα, a Kras sequence was inserted after Ypet to link the whole biosensor to the non-lipid rafts regions on the plasma membrane^52^, to create Kras-sl-RhoGDIα (Fig. 1b). A Lyn sequence was added behind the ECFP to link the biosensor to lipid rafts^52^, giving Lyn-sl-RhoGDIα (Fig. 1b). These two membrane biosensors reflect the binding degree of RhoGDIα and Rho GTPases, respectively, on different positions of the membrane.

All biosensors mentioned above were constructed into pcDNA3.1(+) plasmids for expression in HeLa cells, and biosensor sequences of sl-RhoGDIα and R66E-sl-RhoGDIα were also inserted into a BL21 plasmid to produce and purify proteins for spectrum analysis and western blot in vitro.

### Cell culture and transient transfection

Before transfection, HeLa cells were cultured with the high glucose version of Dulbecco's modified Eagle medium containing 10% fetal bovine serum, 2 mmol per L L-glutamine, 100 units/ml penicillin and 100 mg/ml sodium pyruvate (GIBCO BRL). Lipofectamin 3000 was chosen as the transfection reagent to transfect different DNA plasmids into cells. Cells were passed onto fibronectin-coated cover slips after transfection for 24 h and cultured with 0.5% FBS for 12 h before laminar flow application.

### Flow systems

Laminar flows were provided by a classic parallel-plate flow chamber, modified to fit for dynamic observations under a FRET microscope^19^. Separated HeLa cells were seeded on a glass slide, which was covered by a silicone gasket, and a cover glass. Laminar shear stress was set to 5, 20, and 40 dyn cm^−2^, respectively, by adjusting fluid flow in the chamber^53^. The flow experiments were done at 37 °C with 5% CO_2_ to maintain the pH at 7.4.

### Microscope image acquisition

The microscope image acquisition set-up contained an inverted microscope (Nikon Eclipse Ti Se-ries, Ti-Fl Epi-fl/1) and a cold CCD (Evolve^TM^512, Photometrics). All fluorescence images were acquired on an isolated single cell by MetaFluor software (Universal Imaging) once in every 60s and arranged in chronological order beginning from 001. Images of different channels were created by MetaMorph software (Universal Imaging) for FRET ratio images. The excitation and emission wavelengths of ECFP are 420 and 475 nm, respectively, and the emission wavelength of Ypet is 535 nm.

### Image analysis

A software package using Matlab (Mathworks; Natick, MA) was developed to rapidly analyze the spatio-temporal fluorescence data, which contains three different sections to allow pre-treatment and polarity analysis. First, all fluorescence images are read from two channels of one sample, including parameters indicating the direction of shear stress and the time points when laminar flow was applied. Fluorescence intensity from the four corners of the images was averaged to set the background, which must be subtracted before image quantification and analysis. After filtering speckle and edge recognition, ratio images showing FRET efficiency are achieved by calculating the specific value of FRET/ECFP. The average ratio of whole cell changing with time is shown by a linechart to analyze how shear stress affects the affinity of RhoGDIα and switch II. Second, polarity analysis is processed using ratio images. Single cells in the ratio images are divided into 50 parts, on average, of equal width along the direction of laminar flow. The first part was numbered as 1 to represent the downstream, and the last as 50 to represent the upstream. The percentage of fluorescence intensity in each part from the whole cell is calculated to represent the binding level of local RhoGDIα-Rho GTPases (Supplementary Figure 9). Third, data are combined with continuous time points, and the spatial and temporal changes of RhoGDIα are normalized and drawn into a three-dimensional graph.

### Statistical analysis

All the ratio data were normalized by their basal levels before stimulation in the same cell. Statistical analysis was performed by using a two-tailed t-test function contained in the Excel software (Microsoft) to evaluate the statistical difference between groups. A significant difference was determined by the *p*-value (<0.05). To decrease the discreteness caused by choosing a single region, the first five regions were chosen and averaged at each time point to represent downstream of the cell and the last five regions represented the upstream. When a statistically significant difference existed (compared by two-tailed *t*-test) between the upstream and downstream values at 30 min by using a two-tailed *t*-test, it indicated polarity is present. All means involved in the manuscript are modified by standard deviation.

### Code availability

MATLAB source code for image processing is provided as Supplementary Software 1.

## Electronic supplementary material

Supplementary Software 1

Supplementary Data 1

Supplementary Data 2

Supplementary Data 3

Descriptions of Additional Supplementary Files

Supplementary Information

Supplementary Movie 1

Supplementary Movie 2

Supplementary Movie 3

Supplementary Movie 4

Supplementary Movie 5

Supplementary Movie 6

Supplementary Movie 7

Supplementary Movie 8

Supplementary Movie 9

Supplementary Movie 10

Supplementary Movie 11

Supplementary Movie 12

Supplementary Movie 13

Supplementary Movie 14

Supplementary Movie 15

Supplementary Movie 16

Supplementary Movie 17

Supplementary Movie 18

Supplementary Movie 19

Supplementary Movie 20

Supplementary Movie 21

## Data Availability

The data supporting the study that are not provided in the manuscript and Supplementary Data 1–3 are available from the corresponding author on reasonable request.
