# Supplementary material for: FRET biosensor allows spatio-temporal observation of shear stress-induced polar RhoGDIα activation
Source: Commun Biol. 2018 Dec 10;1:224. doi: 10.1038/s42003-018-0232-2 (PMC6288100; doi:10.1038/s42003-018-0232-2)
Supplement: Supplementary file 5 — Descriptions of Additional Supplementary Files [file 42003_2018_232_MOESM5_ESM.docx]

Supplementary Movie 1. The sl-RhoGDIα biosensor in HeLa cells under shear stress of 5 dyn cm­^-2

Supplementary Movie 2. The Kras-sl-RhoGDIα biosensor in HeLa cells under shear stress of 5 dyn cm­­^-2

Supplementary Movie 3. The Lyn-sl-RhoGDIα biosensor in HeLa cells under shear stress of 5 dyn cm­­^-2

Supplementary Movie 4. The sl-RhoGDIα biosensor in HeLa cells under shear stress of 20 dyn cm­­^-2

Supplementary Movie 5. The Kras-sl-RhoGDIα biosensor in HeLa cells under shear stress of 20 dyn cm­­^-2

Supplementary Movie 6. The Lyn-sl-RhoGDIα biosensor in HeLa cells under shear stress of 20 dyn cm­­^-2

Supplementary Movie 7. The sl-RhoGDIα biosensor in HeLa cells under shear stress of 40 dyn cm­­^-2

Supplementary Movie 8. The Kras-sl-RhoGDIα biosensor in HeLa cells under shear stress of 40 dyn cm­­^-2

Supplementary Movie 9. The Lyn-sl-RhoGDIα biosensor in HeLa cells under shear stress of 40 dyn cm­­^-2

Supplementary Movie 10. The Lyn-sl-RhoGDIα biosensor in HeLa cells under shear stress of 20 dyn cm­­^-2 after pretreatment with 45 mmol/l benzol alcohol for 15 min.

Supplementary Movie 11. The Lyn-sl-RhoGDIα biosensor in HeLa cells under shear stress of 20 dyn cm­­^-2 after pretreatment with 0.1 mmol/l cholesterol for 3 h.

Supplementary Movie 12. The Lyn-sl-RhoGDIα biosensor in HeLa cells under shear stress of 20 dyn cm­­^-2 after pretreatment with 5 μmol/l ML-7 for 1 h.

Supplementary Movie 13. The Lyn-sl-RhoGDIα biosensor in HeLa cells under shear stress of 20 dyn cm­­^-2 after pretreatment with 2 μmol/l CytoD for 1 h.

Supplementary Movie 14. The Lyn-sl-RhoGDIα biosensor in HeLa cells under shear stress of 20 dyn cm­­^-2 after pretreatment with 1 μmol/l NOCO for 1 h.

Supplementary Movie 15. The Lyn-sl-RhoGDIα biosensor in HeLa cells under shear stress of 20 dyn cm­­^-2 after pretreatment with 50 mmol/l PP1 for 30 min.

Supplementary Movie 16. The Kras-sl-RhoGDIα biosensor in HeLa cells under shear stress of 20 dyn cm­­^-2 after pretreatment with 45 mmol/l benzol alcohol for 15 min.

Supplementary Movie 17. The Kras-sl-RhoGDIα biosensor in HeLa cells under shear stress of 20 dyn cm­­^-2 after pretreatment with 0.1 mmol/l cholesterol for 3 h.

Supplementary Movie 18. The Kras-sl-RhoGDIα biosensor in HeLa cells under shear stress of 20 dyn cm­­^-2 after pretreatment with 5 μmol/l ML-7 for 1 h.

Supplementary Movie 19. The Kras-sl-RhoGDIα biosensor in HeLa cells under shear stress of 20 dyn cm­­^-2 after pretreatment with 2 μmol/l CytoD for 1 h.

Supplementary Movie 20. The Kras-sl-RhoGDIα biosensor in HeLa cells under shear stress of 20 dyn cm­­^-2 after pretreatment with 1 μmol/l NOCO for 1 h.

Supplementary Movie 21. The Kras-sl-RhoGDIα biosensor in HeLa cells under shear stress of 20 dyn cm­­^-2 after pretreatment with 50 mmol/l PP1 for 30 min.

Supplementary Software 1: the code used to visualize the polarity and written by Matlab.

Supplementary Data 1: the time-ratio data of overall after normalized, containing means and standard deviations. The data used to calculate the mean and the standard deviation were the averaged overall ratio of different samples after normalization, corresponding to the variable *ave_normalized* in Supplementary Software 1. To the same sample, the software calculated the ratio of FRET channel image to ECFP channel image and ratio per unit area of the whole sample as variable *ave*. Shear stress is applied to samples at the zero point. The normalization is based on the data at zero point to get the variable *ave_normalized.* All experimental conditions are marked as the sheet names.

Supplementary Data 2: the temporal-spatial data of ratio percentage after normalization. The data showed the averaged ratio percentage in each region at each time point after normalization, corresponding to the variable *in2* in Supplementary Software 1 and related to Supplementary Figure 9. The ratio of FRET channel image to ECFP channel image and the whole cell was divided into 50 regions with equal length along the direction of shear stress by the software. The first region was downstream, and the fiftieth region was upstream. The ratio per unit area in each region was divided by the ratio per unit area of the whole and arranged in chronological order forms the variable *inn3*. Shear stress is applied to samples at the zero point. The normalization is based on the data at zero point and achieves the variable *in2*. Experimental conditions are marked as the sheet names.

Supplementary Data 3: the ratio from upstream and downstream of samples under different conditions after normalization. The data showed the normalized ratio percentage at downstream and upstream regions at the thirtieth minute, related to Supplementary Figure 9 and Supplementary data 2. The downstream regions contained region 1 to region 5 while the upstream region contained region 46 to region 50.
