# Supplementary material for: FRET biosensor allows spatio-temporal observation of shear stress-induced polar RhoGDIα activation
Source: Commun Biol. 2018 Dec 10;1:224. doi: 10.1038/s42003-018-0232-2 (PMC6288100; doi:10.1038/s42003-018-0232-2)
Supplement: Supplementary file 6 — Supplementary Information [file 42003_2018_232_MOESM6_ESM.pdf]

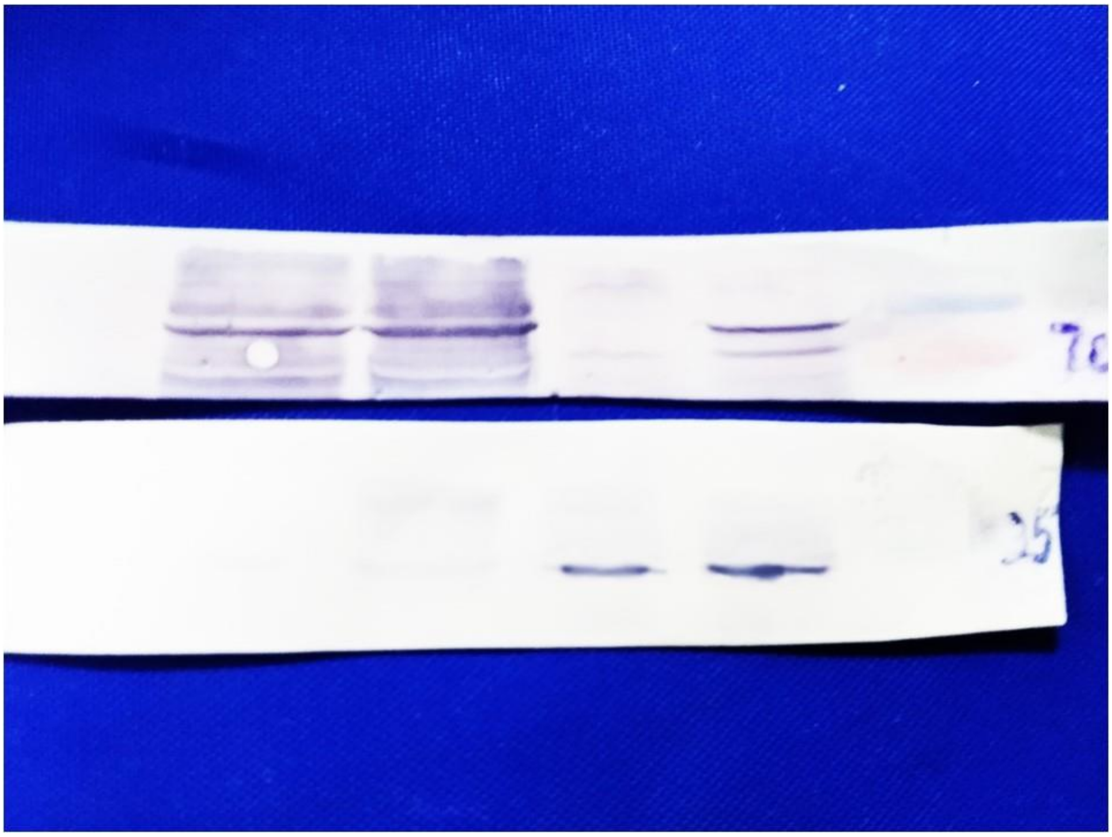

Supplementary Figure 1. The western blot image.

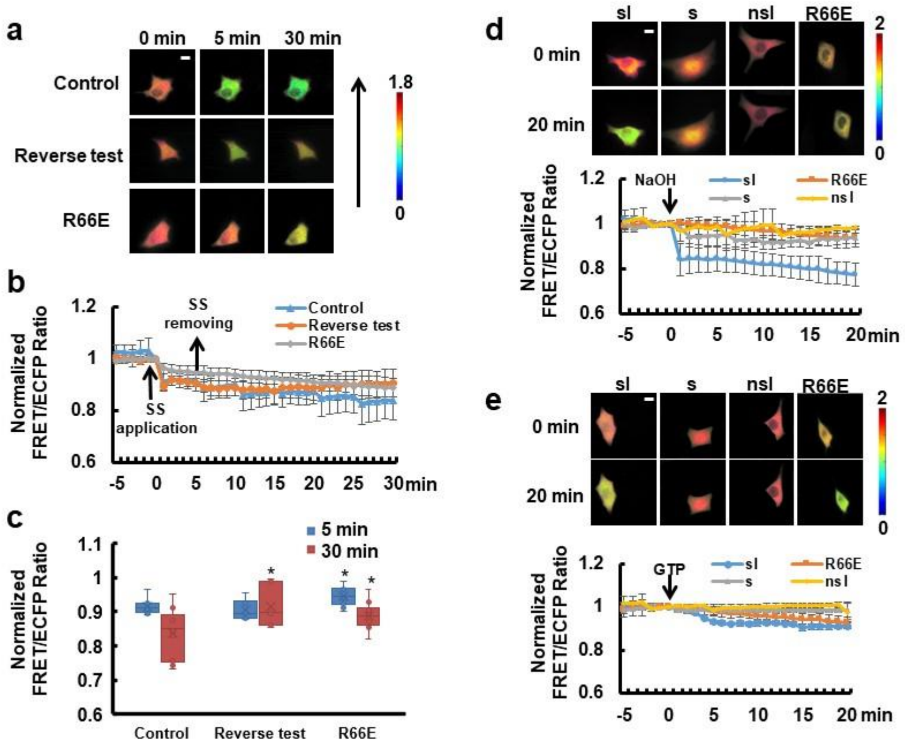

Supplementary Figure 2, related to Fig. 1. The additional verification experiments of biosensors. a) The living cell images upon 20 dyn cm^-2 of shear stress. Cells in control group and reverse test (n=6) are transfected with sl-RhoGDIα biosensor. Cells in R66E group (n=14) are transfected with R66E-sl-RhoGDIα biosensor. The scale bar is 10 μm. b) The dissociation of the RhoGDIα-Rho GTPases complex for reverse test of sl- RhoGDIα biosensor and R66E mutant biosensor. The shear stress is applied at 0 min and removed at 5 min in reverse test but remains to 30 min for control group and R66E group. c) The binding degree at 5 min and 30 min. * represents an obvious difference compared to control group. d) Testing biosensors with NaOH. Under 2 mmol/l NaOH stimulation, the FRET ratio decreases more in cells transfected with sl-RhoGDIα(n=8) than R66E-sl-RhoGDIα(n=12), s-RhoGDIα(n=8) and nsl-RhoGDIα(n=6) biosensor. The scale bar is 10 μm. e) Testing biosensors with GTP. Under 10 μmol/l GTP stimulation, the FRET ratio decreases more in cells transfected with sl-RhoGDIα(n=5) and R66E-sl-RhoGDIα(n=8) than s-RhoGDIα(n=5) and nsl-RhoGDIα(n=6) biosensor. The scale bar is 10 μm.

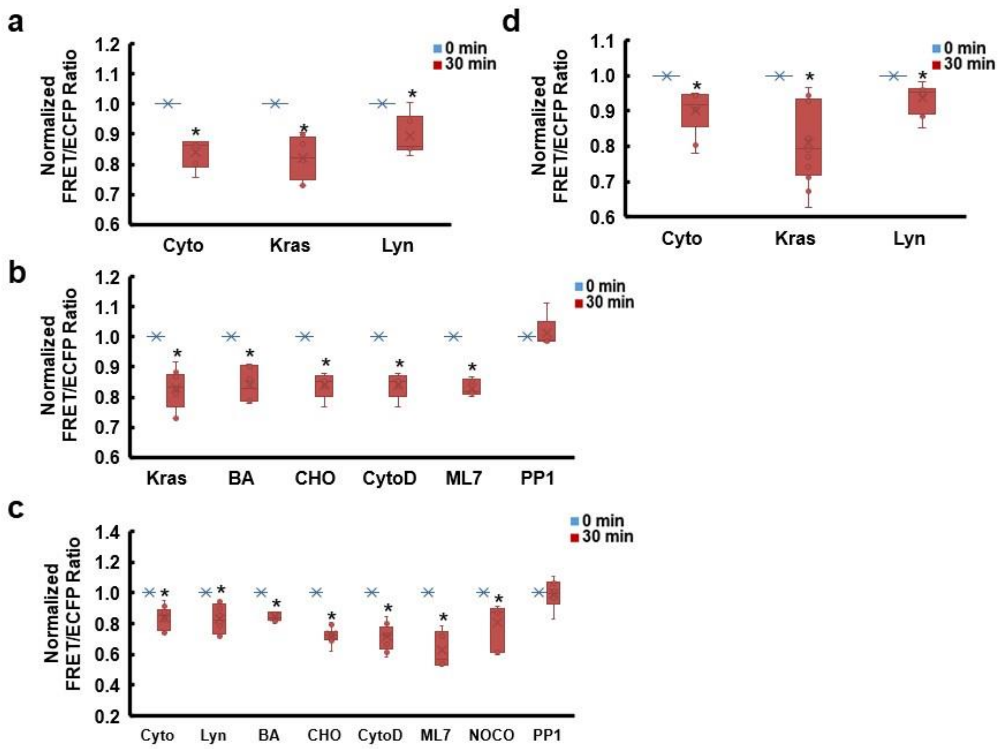

Supplementary Figure 3. The comparison of overall FRET ratio between 0 min and 30 min. a) The comparison of cells transfected with three biosensors respectively upon 5 dyn cm^-2 of shear stress. b) The comparison of cells transfected with Kras-sl-RhoGDIα biosensor upon 20 dyn cm^-2 of shear stress, with or without pretreatments. c) The comparison of cells transfected with Kras-sl-RhoGDIα and sl- RhoGDIα biosensor respectively upon 20 dyn cm^-2 of shear stress. Thereinto, cells transfected with Lyn-sl-RhoGDIα are also pretreated by drugs. d) The comparison of cells transfected with three biosensors respectively upon 40 dyn cm^-2 of shear stress. *represents $p<0.05$.

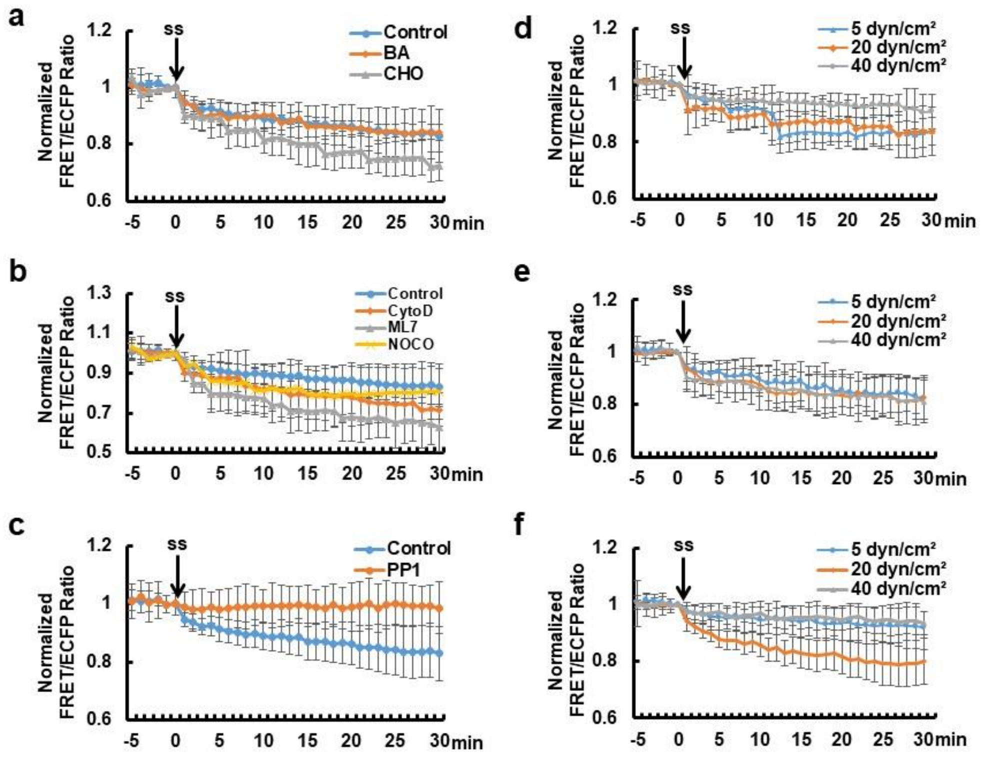

Supplementary Figure 4, related to Fig. 2-Fig. 7. The comparison of RhoGDIα-Rho GTPases complex affinity under different conditions. a) The effect of shear stress on the binding degree of RhoGDIα and Rho GTPases when membrane is enhanced or inhibited. b) The effect of shear stress on the binding degree of RhoGDIα and Rho GTPases when cytoskeleton is disturbed. c) The binding degree of RhoGDIα and Rho GTPases under laminar flow with inhibition of Src. d) The dissociation of the RhoGDIα-Rho GTPases complex under different magnitudes of shear stress in cytoplasm over time. e) The dissociation of the RhoGDIα-Rho GTPases complex under different magnitudes of shear stress in non-lipid regions on membrane over time. f) The dissociation of the RhoGDIα-Rho GTPases complex under different magnitudes of shear stress in lipid rafts over time.

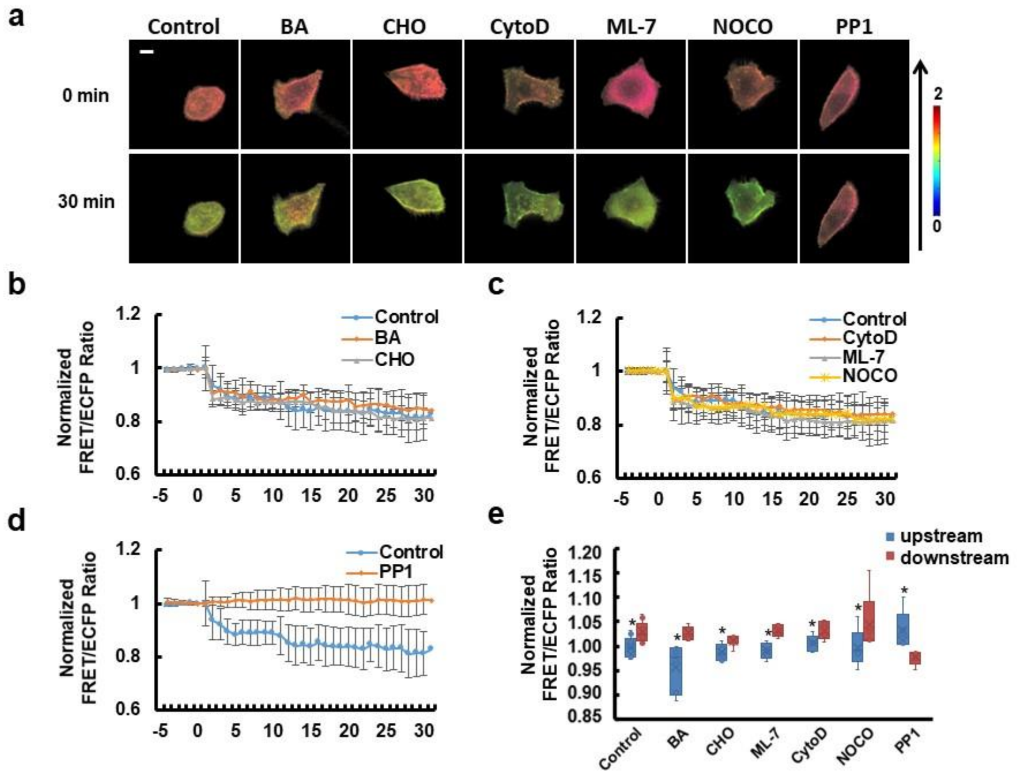

Supplementary Figure 5. The results depending on Kras-based biosensor upon shear stress. a) Living cell images with Lyn-sl-RhoGDIα under 20 dyn cm^-2 of shear stress. The scale bar is 10 μm. b) The effect of shear stress on the binding degree of RhoGDIα and Rho GTPases when membrane is enhanced or inhibited (n_{BA}=6, n_{CHO}=7). c) The effect of shear stress on the binding degree of RhoGDIα and Rho GTPases when cytoskeleton is disturbed (n_{CytoD}=5, $n_{ML\text{-}7}$=5, n_{NOCO}=5). d) The binding degree of RhoGDIα and Rho GTPases under laminar flow with inhibition of Src(n_{pp1}=5). e) The FRET/ECFP ratio comparison of upstream to downstream, after normalization. * represent there is an obvious difference between upstream and downstream.

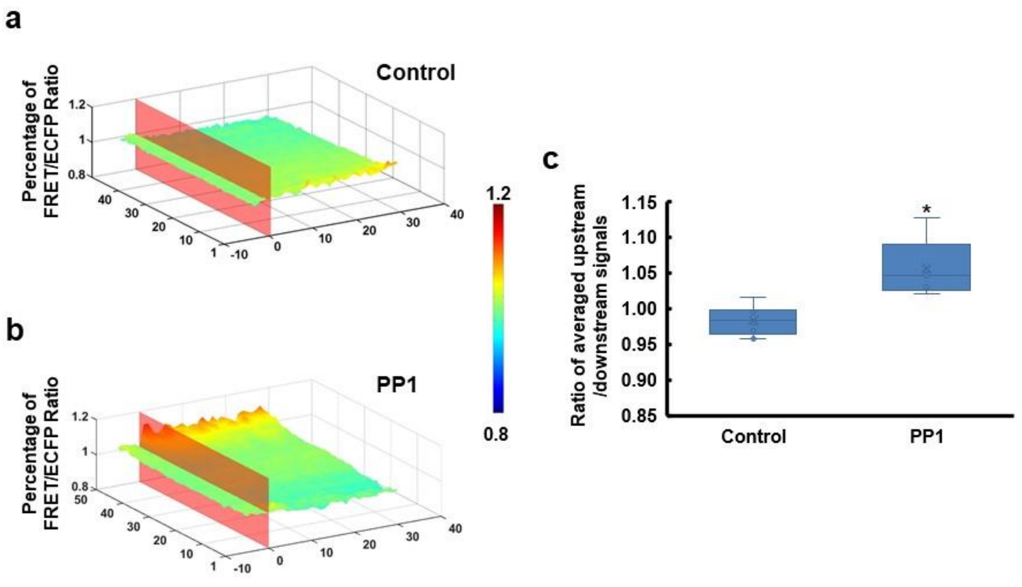

Supplementary Figure 6. The affinity distribution of RhoGDIα and Rho GTPases under shear stress when Src is inhibited. a) The result of control group. b) The result of Src group. c) The comparison results of upstream/downstream in FRET ratio from different groups. * represent there is an obvious difference compared to control group.

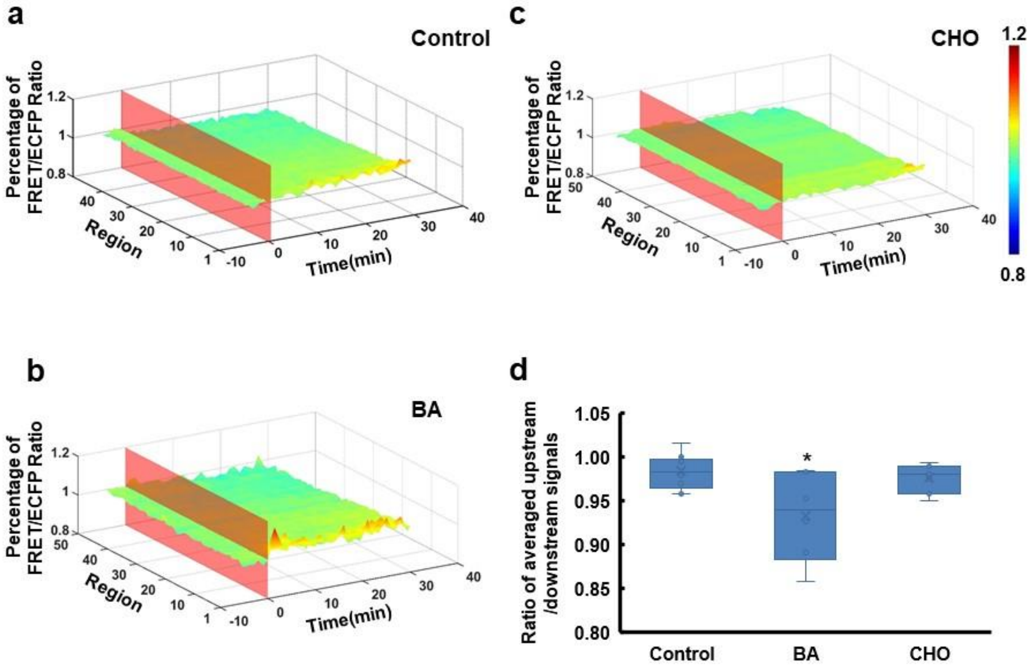

Supplementary Figure 7. The affinity distribution of RhoGDIα and Rho GTPases under shear stress when membrane fluidity is disturbed. a) The result of control group. b) The result of BA group. c) The result of CHO group. d) The comparison results of upstream/downstream in FRET ratio from different groups. * represent there is an obvious difference compared to control group.

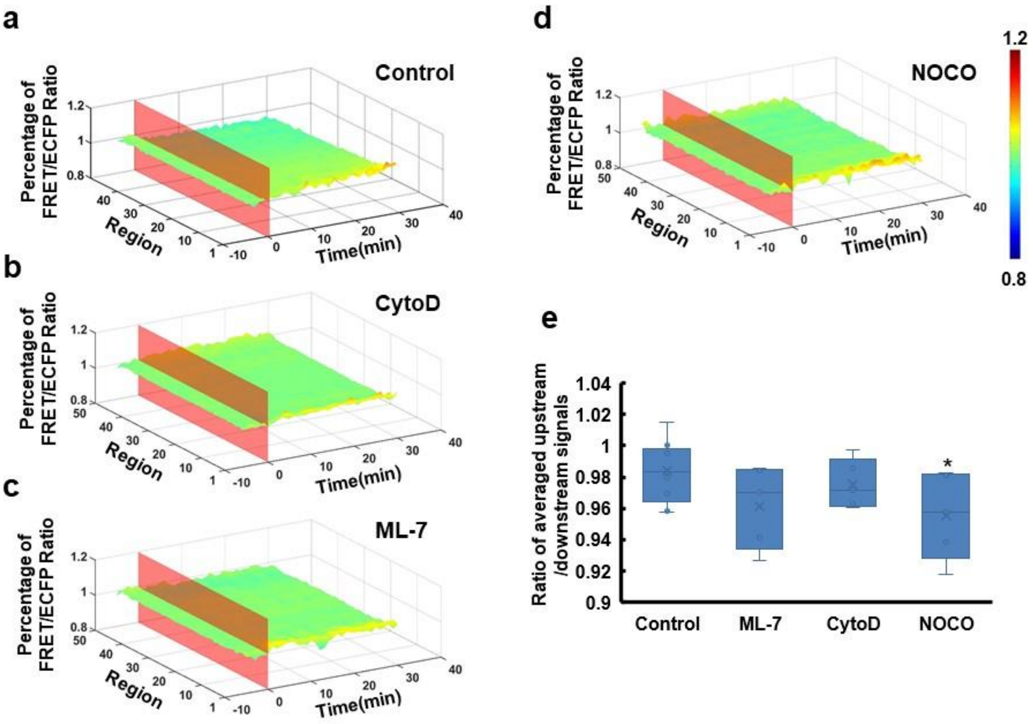

Supplementary Figure 8. The affinity distribution of RhoGDIα and Rho GTPases under shear stress when cytoskeleton is disturbed. a) The result of control group. b) The result of Cyto group. c) The result of ML-7 group. d) The result of NOCO group. e) The comparison results of upstream/downstream in FRET ratio from different groups. * represent there is an obvious difference compared to control group.

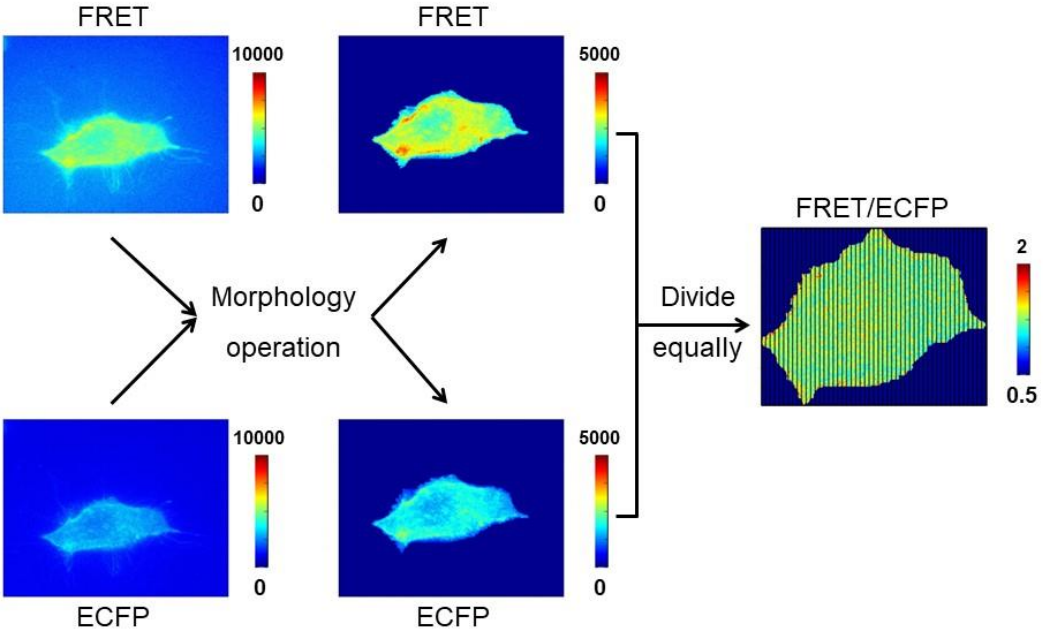

Supplementary Figure 9, related to Fig. 2 - Fig. 7. The procedure for image processing**.**

Supplementary Table 1 the p value of overall value between 0 min and 30 min on lipid raft

Lyn	ML-7	CytoD	NOCO	BA	CHO	PP1
0.001	0.002	7.776E-06	0.001	8.265E-07	3.235E-08	0.865

Data for t-test was the ratio per unit area of the whole cell after normalization.

Supplementary Table 2 the p value of overall value between 0 min and 30 min at non-lipid raft regions

Kras	ML-7	CytoD	NOCO	BA	CHO	PP1
0.013	0.001	0.001	0.001	0.001	1.48279E-05	0.670

Data for t-test was the ratio per unit area of the whole cell after normalization.

Supplementary Table 3 the p value of different subcellular locations at 30min

	5 dyn cm^-2	20 dyn cm^-2	40 dyn cm^-2
Cyto-Kras	0.618	0.745	0.030
Cyto-Lyn	0.012	0.820	0.209
Kras-Lyn	0.007	0.880	0.003

Data for t-test was the ratio per unit area of the whole cell after normalization.

Supplementary Table 4 the p value of overall at 30min compared to control

	ML-7	CytoD	NOCO	BA	CHO	PP1
Kras	0.844	0.696	0.844	0.696	0.919	0.033
Lyn	0.005	0.020	0.715	0.722	0.007	0.006

Data for t-test was the ratio per unit area of the whole cell after normalization.
